# Influence of Clinical Aspects and Genetic Factors on Feline HCM Severity and Development

**DOI:** 10.3390/vetsci11050214

**Published:** 2024-05-13

**Authors:** Victoria Korobova, Yulia Kruglova

**Affiliations:** Moscow State Academy of Veterinary Medicine and Biotechnology Named after K.I. Skryabin, 23 Akademika Skryabina str., Moscow 109472, Russia

**Keywords:** hypertrophic cardiomyopathy (HCM), cats, *MYBPC3*, DNA testing, p. A31p mutation, clinical diagnostics

## Abstract

**Simple Summary:**

Hypertrophic cardiomyopathy (HCM) is the most common feline heart pathology, which is characterized by thickening of the ventricular walls. This disease is genetic in nature and often occurs in cats of certain breeds such as Maine Coon, Persian, Ragdoll, and Norwegian Forest. In this study, we attempted to identify the significance of determining genetic predisposition to this disease using DNA tests. We found that in homozygous cats, the disease manifests itself earlier and its progression is more severe than in heterozygous cats. Since the disease is often asymptomatic, we compared the quality of various diagnostic methods and clinical aspects for identifying HCM at an early stage.

**Abstract:**

Hypertrophic cardiomyopathy (HCM), which is associated with thickening of the left ventricular wall, is one of the most common heart pathologies in cats. This disease has a hereditary etiology and is primarily related to mutations in the *MYBPC3* and *MYH7* genes. This study aims to determine the effect of the presence of heterozygosity or homozygosity for the p. A31P mutation (c.91G>C) in the *MYBPC3* gene in cats (Maine Coon) of different ages referring to the HCM severity and development, and to compare echocardiographic data and various clinical aspects for the most objective detection of disease in cats of different breeds. The incidence of HCM was 59% of the 103 cases of heart disease in cats in this study. In 23 cats diagnosed with HCM, cats heterozygous for the mutation accounted for 34%, and homozygous cats accounted for 26%. Cats homozygous for this mutation had moderate to severe HCM, suggesting an association with high penetrance of HCM and a significant risk of cardiac death in this group. The penetrance of the heterozygous type was lower than that of the homozygous genotype. This study also indicates that HCM has some age-related penetrance. The disease did not occur in the study group of cats aged up to 1 year, whereas at the age of 7 and older, the percentage of animals diagnosed with HCM was the highest and amounted to 44.3% of the total number of studied cats with HCM.

## 1. Introduction

Hypertrophic cardiomyopathy (HCM) is one of the most common heart diseases in cats. It is a genetically heterogeneous myocardial disease with an autosomal dominant mode of inheritance, i.e., the presence of even one mutant allele is sufficient for the development of this disease. Genetic heterogeneity, among other things, refers to mutations in one gene that phenotypically exhibit incomplete penetrance, i.e., cause different clinical manifestations of disease severity. The mechanism of this pathology at the cellular level is a disruption of the myocardial structure, which leads to the loss of the parallel arrangement of myocytes [1].

Disruption of the sarcomere protein structure (MBPC) alters the molecular process of muscle contraction and leads to activation of sarcomere replication, which increases myocyte width and, consequently, wall thickness (hypertrophy) [2].

The HCM consequence is diastolic dysfunction, a decrease in the heart’s ability to fill normally during diastole, which leads to increased pressure in the left atrium and the subsequent development of congestive heart failure.

In humans, approximately 1400 mutations present in at least 13 genes are known to cause HCM [3]. The overwhelming majority of mutations are in the genes encoding the beta-cardiac/slow skeletal myosin heavy chain (*MYH7*) and cardiac myosin-binding protein C (*MYBPC3*). Mutations encoding myosin regulatory light chains (*MYL2* and *MYL3*), α-cardiac actin (*ACTC1*), α-tropomyosin, troponin I (*TNNI*), troponin T (*TNNT*), and titin are less common [4].

This study aimed to determine the effect of heterozygosity or homozygosity for the p. A31P mutation (c.91G>C) in the *MYBPC3* gene in cats (Maine Coon) of various ages on HCM severity and development, and to compare echocardiographic data and various clinical aspects for the most objective identification of HCM in cats of different breeds.

According to the European Society of Cardiology (ESC) classification, adapted for cats, phenotype-based cardiomyopathies are classified into hypertrophic cardiomyopathy (HCM), restrictive cardiomyopathy (RCM), dilated cardiomyopathy (DCM), unclassified cardiomyopathy (UCCM), and arrhythmogenic right ventricular cardiomyopathy (ARVC) [5].

To describe the clinical consequences of cardiomyopathy in affected cats, a system adapted from the American Heart Association (AHA) and American College of Veterinary Internal Medicine (ACVIM) cardiac staging systems is proposed to provide a basis for prognosis and therapeutic decision making.

Stage A includes cats that are predisposed to cardiomyopathy but have no evidence of myocardial disease. Stage B describes cats with cardiomyopathy but no clinical signs. Stage B is subdivided into stage B1: cats at low risk for imminent congestive heart failure (CHF) or arterial thromboembolism (ATE), and stage B2: cats at higher risk for CHF or ATE. Atrial size is a crucial prognostic marker that can be used to categorize cats with subclinical cardiomyopathy into low-risk (B1) and high-risk (B2) cats. The greater the left atrium (LA) enlargement, the higher the risk of CHF and ATE [5].

HCM is the most commonly encountered cardiomyopathy in cats during echocardiography (approximately 60% of cases). RCM and UCCM phenotypes account for 20–30%. Historically, 1 in 500 people have HCM, although more recent evidence suggests that the rate may be much higher (1 in 200 to 1 in 70) [6]. In cats, this rate is closer to 1 in 7 based on echocardiographic screening for HCM [7].

Two out of six genetic causes of HCM [8] identified to date in domestic cats are associated with the same gene, *MYBPC3*, which encodes myosin-binding protein C (cMyBP-C). It is a muscle regulatory protein that influences the strength and speed of heart contraction and contributes to both diastolic and systolic function and the cardiac ability to increase contractility in response to inotropic stimuli [9,10]. In cats, missense mutations in the *MYBPC3* gene cause substitutions of individual amino acids in different domains of the affected proteins. However, as with human *MYBPC3* mutations, it is currently unknown whether these mutations cause this disease by disrupting the normal function of the cMyBP-C protein, affecting its stability, and resulting in disruption of other cellular processes.

The *MYBPC3* gene (located on chromosome D1 in cats) is more than 21 kbp in size. and consists of 35 exons (the size of 1 exon is approximately 4.5 kbp), of which 34 encode amino acids. cMyBP-C is a sarcomeric protein comprising 1173 amino acids located within the transverse A-band of the sarcomere and is associated with titin and thick α-myosin filaments. cMyBP-C is a member of the immunoglobulin (Ig) superfamily of proteins, consisting of 11 modular domains folded into compact β-sheets with homology to either Ig-like or fibronectin (Fn)-like domains. Domains are sequentially numbered C0–C10, starting from the N-terminus of the protein. The p. A31P mutation found in Maine Coon cats places an altered amino acid near the N terminus of the cMyBP-C protein in the C0 region, which is the first Ig-like domain of cMyBP-C. However, the p. R820W mutation found in Ragdoll cats occurs closer to the center of the molecule in the C6 domain region. An unstructured linker rich in proline and alanine residues is located between domains C0 and C1, and a partially structured linker (about 100 residues), called the M domain, is located between C1 and C2. The M domain is a key regulatory subunit of the cMyBP-C protein, which is phosphorylated by protein kinases (e.g., protein kinase A) after β-adrenergic stimulation [11,12].

Various domains and linkers of the cMyBP-C protein are thought to influence different functions of cMyBP-C by interacting with other sarcomeric proteins. For example, domains C8–C10 anchor cMyBP-C to myosin and titin in the thick filament, and these interactions explain the characteristic localization of cMyBP-C during electron microscopy as a series of 7–9 discrete bands (corresponding to approximately 1 molecule of cMyBP-C for every 9 myosin molecules) in each half of the sarcomere [13]. Domains C1–M–C2 are also associated with myosin, but in a different segment of the β-myosin heavy chain. Thus, these N-terminal domains of the cMyBP-C protein are thought to influence the ability of myosin to interact with actin and thereby inhibit or enhance contraction. In addition, the same N-terminal domains, together with C0 (a unique domain of the cardiac isoforms of the MyBP-C protein), directly interact with actin and may promote contraction activation through a novel mechanism that shifts tropomyosin on the thin filament. Thus, the activating effects of the N-terminal domains of the cMyBP-C protein may explain the ability of cMyBP-C to maintain force during systole and prolong the systole ejection phase [14].

Since the p. A31P mutation occurs in the C0 domain region, it may impair the ability of C0 to interact with actin and/or thin filament, thereby acting as a toxic polypeptide affecting systolic or diastolic function. In compliance with this idea, three HCM-causing mutations in humans that occur in the M domain of the cMyBP-C protein (which is also associated with actin) reduce the level of binding of cMyBP-C to actin, whereas another HCM-causing mutation increases actin binding. Alternatively, since C0 also interacts with the myosin regulatory light chain (RLC), the p. A31P mutation may disrupt the interaction with the RLC and thus alter the ability of myosin to generate force. Interestingly, very little is known about the “middle” domains of the cMyBP-C protein (C3 to C7). Thus, the functional significance of the C6 domain, which contains the abnormal amino acid created by the p. R820W mutation in Ragdoll cats and humans, is currently unknown [15].

The idea that the p. A31P gene mutation can create a protein that functions as a toxic polypeptide, further supported by the observation that the cMyBP-C mutant protein is properly incorporated into muscle sarcomeres. An antibody specific for the p. A31P missense mutation recognizes the abnormal protein in the myofibrils of heterozygous and homozygous affected cats [16].

Since the p. A31P mutation affects the folded structure of C0, increased degradation of misfolded mutant cMyBP-C may lead to secondary cellular stress effects that contribute to pathogenesis.

## 2. Materials and Methods

This research was conducted in a private veterinary clinic, two specialized genetic laboratories: “Chance” and “Vet Genomics”, and at the premises of the Department of Disease Diagnostics, Therapy, Obstetrics and Animal Reproduction of the Moscow State Academy of Veterinary Medicine and Biotechnology—MVA named after K.I. Skryabin.

### 2.1. The First Phase

In the first phase of the research, 103 cats (12 different breeds) diagnosed with heart disease were studied from 2021 to 2023 to determine the incidence of HCM among heart diseases and identify the risk group depending on breed, age, and sex. All cats belonged to owners living in Russia and were patients of the cardiology department of a private veterinary clinic. All cases of heart disease were identified by searching the electronic records of feline patients observed at several local veterinary centers. Cats were included in the study if diagnosed by a board-certified cardiologist based on 2D or M-mode echocardiography or both, and if follow-up information was available. Each cat’s medical records were reviewed, and the following data were included: date of birth, sex, breed, date of the first visit to a cardiologist, and the calculated age at the time of that visit. Physical examination results, systolic blood pressure, and total serum thyroxine concentrations were also included.

### 2.2. The Second Phase

In the second phase, 23 cats, aged 1.6–12.5 years, weighing 3.5–10.2 kg, with echocardiographic signs of HCM and unknown mutation status in the *MYBPC3* gene, were studied between October 2022 and April 2023. For this study, owners of cats observed at a local veterinary center with a confirmed diagnosis of HCM were offered free heart screenings. The owners of 23 cats provided written consent for their cats to participate in the study. At each visit, the following data were collected: age, body weight, heart rate (HR), presence of murmur, gallop or arrhythmia, and presence of clinical symptoms.

All cats underwent echocardiography using the Doppler method in accordance with the recommendations, chest radiography, ECG, and general clinical and biochemical blood tests, the NT-proBNP value was >99 pmol/L. Five different breeds were represented: Norwegian Forest Cat (*n* = 3), Domestic Shorthair (*n* = 2), Maine Coon (*n* = 14), British Shorthair (*n* = 2), and Persian (*n* = 2). Of these, 15 animals (65%) were castrated males.

Cats with HCM were selected for the research based on several criteria, including a heart murmur, gallop rhythm, or arrhythmia; clinical signs of heart failure or arterial thromboembolism; fainting; and cardiomegaly detected on chest X-ray.

Collection and analysis of anamnestic data according to the cardiological examination plan (presence of cough, shortness of breath, fainting, whether additional examinations (ultrasound, blood test) were performed and when/how often, whether a decrease in activity, lethargy, not typical for this animal, was observed, and a decrease in exercise tolerance, abdominal enlargement, and cyanosis of the tongue and oral mucosa (temporary/permanent). Classification of heart murmurs is the Levine classification.

At this phase, DNA testing was conducted to identify the p. A31P mutation in the *MYBPC3* gene using SNP genotyping in 23 cats with a confirmed diagnosis of HCM (5 different breeds).

### 2.3. Exclusion Criteria

Hyperthyroidism and hypertension were excluded as secondary causes of concentric hypertrophy by measuring blood pressure and total T4. Blood pressure was considered normal if the systolic blood pressure was <150 mmHg. Cats with signs of systemic disease or hyperthyroidism were not included in the study.

Exclusion criteria were any other disease, systemic hypertension, hyperthyroidism or aortic stenosis, dehydration, and examination of cats under general anesthesia, or kidney disease.

Unlike HCM, the cause of secondary hypertrophy development is compensatory in nature. The therapeutic significance of this difference lies in the fact that with secondary hypertrophy, after successful treatment of the primary disease, the thickness of the heart wall can return to normal, whereas HCM is an irreversible condition.

### 2.4. Echocardiographic Studies (23 Cats)

Echocardiographic studies of cats were conducted using a Philips Affiniti 50 ultrasound system (Koninklijke Philips N.V., Eindhoven, The Netherlands). The LV wall thickness was measured using B-mode and M-mode. Measurements of the left atrium were performed in 2D mode in the right parasternal projection at the level of the aortic valve along the short axis in relation to the aortic diameter. Cats were not sedated; they were kept in right and left lateral recumbency. Standard right parasternal long- and short-axis views plus left apical and left cranial views were examined. Signs of SAM were determined using the 2D mode in the right parasternal projection at the level of the left ventricle outflow tract along the long axis. Color Doppler sonography was used to identify the characteristic blood flow jets of LVOT obstruction and mitral regurgitation, and spectral Doppler was used to assess the peak LVOT gradient from the left apical view. HCM was diagnosed in cats when interventricular septal thickening was present and/or the posterior LV wall or the entire LV wall was ≥6 mm thick when measured. At least three measurements were taken in each segment, and the average value of the measurements for each segment was calculated.

Cats with HCM were classified according to echocardiographic findings into one of the following groups: mild (diastolic IVS and LVPW thickness of 6.0–6.5 mm and LA/Ao < 1.5), moderate (IVS and LVPW thickness of 6.5–7.0 mm and LA/Ao < 1.8) and severe (IVS and LVPW thickness > 7.0 mm; and LA/Ao > 1.8).

HCM was classified as severe if the end-diastolic thickness of the left ventricular posterior wall was >7 mm and at least one of the following conditions was present: systolic LV chamber obliteration (failure to detect the presence of a left ventricular cavity at end systole); SAM; dilatation of the left atrium or the presence of a thrombus in the left atrium during echo contrast.

### 2.5. Chest Radiography (23 Cats)

Right lateral chest radiographs were obtained from each cat using a GIERTH HF 500 smove DR veterinary digital radiography (GIERTH X-ray international GmbH, Riesa, Germany). Particular attention was paid to correct positioning of the cats on the radiographic table.

When determining the size of the heart, three factors are considered:

1. Position of the trachea relative to the spinal axis (normally, an angle of at least 30°);
2. The cardiac silhouette measures approximately two intercostal spaces in width on the lateral projection;
3. In the laterolateral projection, the Buchanan cardiac index is the most accurate method for determining the heart size in radiographs.

Normally, the Buchanan index for cats is:

L = T4 × 4.8; B = T4 × 3.0

where L is length; B—width; T4—length of the 4th thoracic vertebral body.

On the dorsoventral projection, the cardiothoracic index (CTI) is used to determine the heart size.

CTI = cardiac silhouette width/chest width ratio = 0.5.

### 2.6. ECG (23 Cats)

Electrocardiograms were recorded using an electrocardiograph built into the ultrasound machine. All cats were fully conscious without sedation during recording. The animals were placed in a right lateral decubitus position, with their limbs held as perpendicular to the body as possible. Surface electrodes made from flattened alligator clips were attached to the skin, and surgical alcohol was used to maintain proper electrical contact. The electrodes were placed in the middle of the caudal side of the elbows and over the patellas of the hind limbs. Standard 6-lead ECGs (leads I, II, III, aVR, aVL, and aVF) were recorded for all cats. Recordings were made at a paper speed of 50 mm/s and an amplitude of 1 cm = 1 mV.

Seven ECG variables that may be related to LA size were measured or calculated: (1) amplitude (mV) and (2) duration (seconds) of the P wave, (3) duration (seconds) of the PR interval, (4) duration of the P wave relative to the PR interval (%), (5) amplitude and duration of the P wave, (6) duration of the descending or terminal part of the P wave as compared to the duration of the ascending part of the P wave, and (7) the value of the mean electrical axis (MEA) of the P wave in the frontal plane.

### 2.7. DNA Testing (23 Cats)

To identify gene mutations, a fully automated sequencing system based on multicolor fluorescent labeling technology was used.

DNA was extracted from peripheral blood using a commercial kit (Illustra Blood Genomic Prep Mini-Spin Kit, Global Life Sciences Solutions USA LLC, Marlborough, MA, USA) in compliance with the manufacturer’s instructions.

Samples were genotyped by direct sequencing of both DNA strands using the ABI Dye Terminator Sequencing Chemistry reagent kit (Applied Biosystems by Thermo Fisher Scientific Baltics UAB, Vilnius, Lithuania) and the Applied Biosystems 310 DNA Analyzer (Applied Biosystems by Thermo Fisher Scientific Baltics UAB, Vilnius, Lithuania) according to the protocols. One amplicon for the p. A31P mutation consisted of 209 nucleotide pairs. It was obtained using the following special primers: F1-5′GAAGCCAAGGTCAGTGGAAG, and R1-5′CCTACGCAGTCATCGCTG. Standard DNA polymerase amplification was performed in a final volume of 30 μL using 33 cycles at an annealing temperature of 58 °C. Electrophoresis was then performed and base pair changes were compared.

### 2.8. Statistical Analysis

Tabular data are presented as mean ± standard deviation. All echocardiographic data were checked visually and for normality of distribution using the Kolmogorov–Smirnov test. Sex, age, presence of concentric hypertrophy in the selected segment (presence or absence on regional HCM), and heart rate were analyzed in a general linear mixed model with multivariate analysis. SPSS Statistics software (Version 13.0, SPSS Inc., Chicago, IL, USA) was used for analysis. Statistical significance was defined as *p* < 0.05.

## 3. Results

### 3.1. First Phase—Incidence of Feline HCM and Risk Groups Depending on Breed, Age and Gender

The results of examining cats (*n* = 103) with cardiovascular disease from 2021 to 2023 are presented in Table 1 and Table 2.

Of the 103 cases (Table 1), 77 were primary cardiomyopathies, 16 were secondary heart diseases, and 10 were congenital pathologies. In a group of 77 cats with cardiomyopathies, HCM *n* = 61, cats with restrictive cardiomyopathy (*n* = 5), cats with arrhythmogenic right ventricular cardiomyopathy *n* = 6, cats with unclassified cardiomyopathy (*n* = 5) were included. Congenital heart pathologies (*n* = 10) included atrial septal defect (*n* = 2), ventricular septal defect (*n* = 3), tricuspid valve dysplasia (*n* = 1), two-chamber right ventricle (*n* = 1), aortic stenosis (*n* = 1), and mitral valve dysplasia (*n* = 2). Secondary heart diseases (*n* = 16) included cardiac neoplasm (*n* = 2), hyperthyroidism (*n* = 12), hypertension (*n* = 2).

According to data from Table 2, of 103 cases of heart disease in cats (of different ages and genders, 12 different breeds), the occurrence of HCM was 59% (*n* = 61) of the total number of cardiomyopathies cases (*n* = 77). In total, out of 103 cases of cats with cardiac disease, the cardiomyopathies group had the highest percentage—74.7% (77 cats out of 103), including HCM.

Table 3 shows the direct relationship between age and the available disease; thus, at the age of 7 or more, the percentage of animals diagnosed with HCM is 44.3%, 34.4% at the age of 4–5 years, and 21.3% at the age of 1–3 years. All cats are castrated.

As can be seen from Table 4, males are most susceptible to HCM; the HCM incidence in them was 67.2%, which is twice as much as this indicator in females (32.8%).

### 3.2. Second Phase—Features of Diagnosing Feline Hypertrophic Cardiomyopathies

From October 2022 to April 2023, a comprehensive examination of 23 cats admitted to the veterinary clinic with signs of hypertrophic cardiomyopathies was conducted.

From the anamneses in two specific cases, a violation of limb motor function was revealed due to obstruction of the collateral arteries by a thrombus. These patients were additionally prescribed thrombolytic therapy.

On cardiac auscultation, all (*n* = 9) cats with SAM had a grade 3/6 heart murmur.

The gallop rhythm was present in 31% of cats with HCM (*n* = 7).

Heart murmurs, mostly of grade 3/6, were present in 60% of cats with HCM (*n* = 14), with 9% of cats (*n* = 2) having a grade 4/6 murmur. In 13% (*n* = 3) of cats, there were no heart murmurs on auscultation.

Hematological analyses of blood samples from cats suffering from hypertrophic cardiomyopathy were performed during the study (Table 5).

The results of blood biochemical study of cats suffering from hypertrophic cardiomyopathy are presented in Table 6.

Median 2D-guided M-mode LVWd and IVSd measurements in cats with HCM were 6.5 mm (IQR 6.0–8.6 mm; range 5.1–8.6 mm) and 6.5 mm (IQR 5.8–7.1; range 5.3–7.1), respectively. Median LA/Ao was 1.13 mm (IQR 1.07–1.29, range 0.9–1.8).

All cats with severe HCM showed increased left atrial diameter and prolonged isovolumic relaxation time (Table 7).

**Figure 1:**
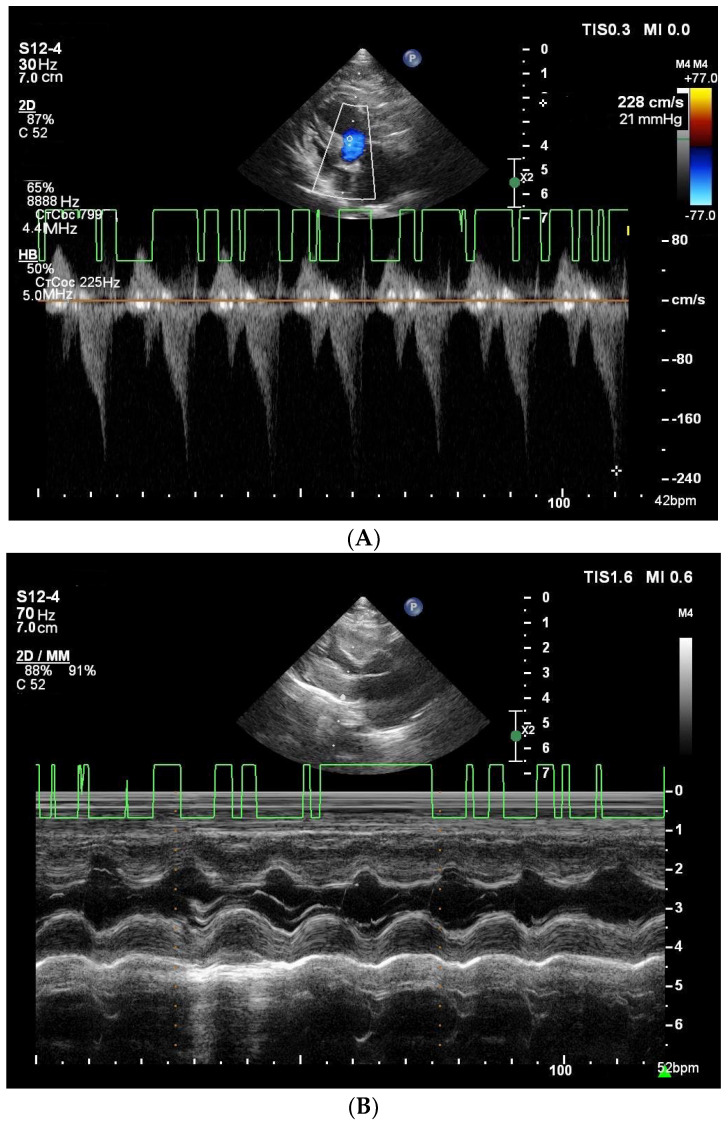

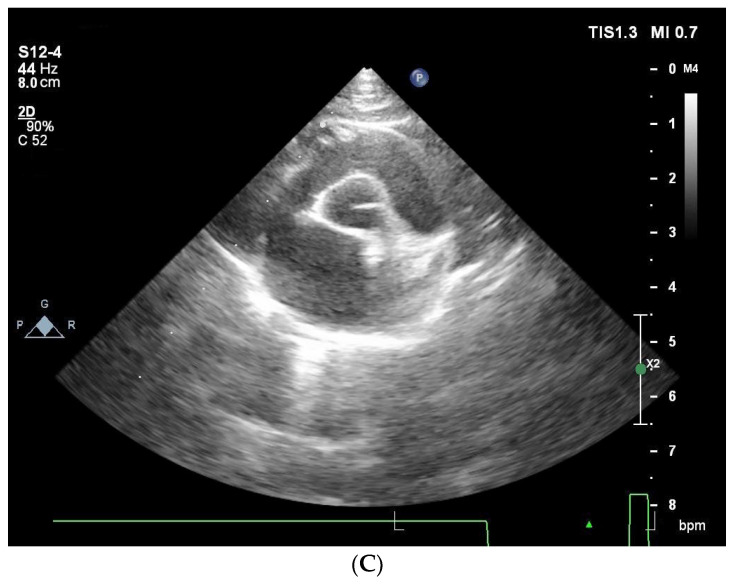
Echocardiographic images (cat, 10 years old, 7.6 kg, Domestic Shorthair), diagnosis: obstructive form of HCM (stage B2), demonstrating anterior systolic mitral motion. (**A**) transmitral flow, IVRT. (**B**) left ventricle in M-mode. (**C**) aorta in relation to the left atrium, dilatation of the left atrium.

Anterior systolic mitral motion was identified in nine cats with severe to moderate HCM (Figure 1).

Chest X-ray examination showed evidence of congestive heart failure (CHF) in three cats. The following abnormalities were found on chest radiographs of cats with HCM: signs of congestive heart failure were indicated by the presence of pleural effusion and/or pulmonary oedema on chest radiographs and/or ascites. Three (13%) cats with severe HCM had signs of pulmonary oedema and two of them (8.7%) had signs of pleural effusion, and only one had radiological signs of ascites. Generalized cardiomegaly was detected in 13 (56.5%) of 23 cats with HCM.

The electrocardiographic study revealed abnormalities in 11 cats (47.8%). The most common findings were left His bundle branch block and a left ventricular enlargement pattern (QRS complexes > 0.04 s and R waves > 0.9 mV).

Seventeen of the 23 cats (73.9%) had at least one supraventricular premature complex, most of which were single isolated complexes. Eight cats (34.7%) had only single ventricular extrasystoles, but six cats (26%) had ventricular tachycardia (five or more complexes in a row).

### 3.3. Relationship between HCM Manifestation and Development in Cats with Identified Missense Mutations in the MYBPC3 Gene

**Figure 2:**
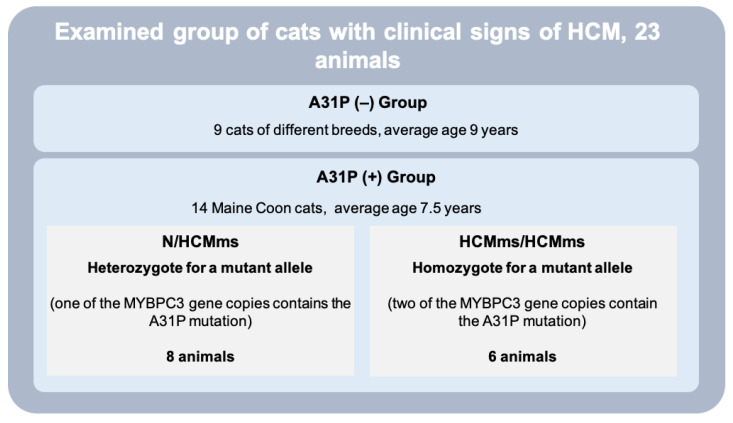
Dependence of HCM on the A31P mutation.

SNP genotyping revealed a change in one base pair (guanine to cytosine) in codon 31 (exon 3) of the *MYBPC3* gene (c. 91G>C). This mutation results in the replacement of a conserved amino acid from alanine (A) to proline (P) (mutation p. A31P). In cats with clinical signs of HCM, this mutation was identified only in Maine Coon cats. Affected cats were either heterozygous (*n* = 8) or homozygous (*n* = 6) for the mutation, i.e., they had one or two affected alleles (Figure 2).

Homozygosity for the p. A31P mutation is associated with high HCM penetrance and a significant risk of cardiac death. Regarding genotypic classification during echocardiography, the values of maximum aortic blood flow velocity were higher in homozygotes.

**Figure 3:**
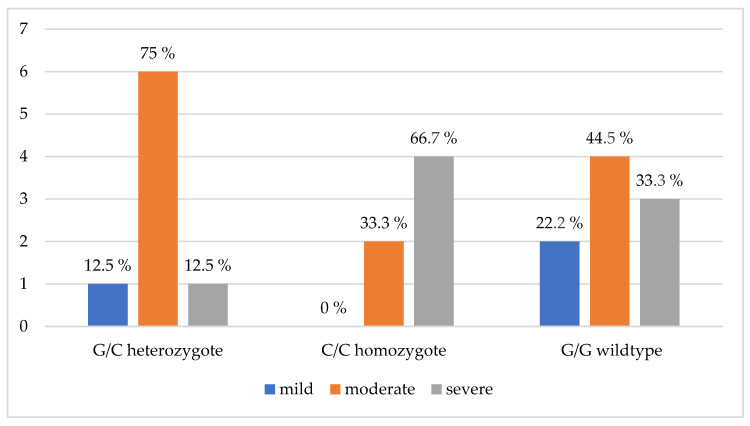
Dependence of HCM severity on the presence of the p. A31P mutation in the *MYBPC3* gene. The drawing was made by the author on the basis of studies conducted in 23 cats diagnosed with HCM; G/C—heterozygous for a mutant allele (one of the copies of the *MYBPC3* gene contains the A31P mutation)—8 animals; C/C—homozygous for a mutant allele (two copies of the *MYBPC3* gene contain the A31P mutation)—6 animals; G/G—homozygous for the normal allele (both copies of the *MYBPC3* gene do not contain the A31P mutation)—9 animals.

All cats participating in this study that were homozygous for this mutation had moderate to severe HCM (Figure 3).

On average, 23 cats with HCM were followed for 3.4 years. Initially (Table 8), a mild degree (stage B1) of HCM was observed in three cats (*n* = 1 heterozygote; *n* = 2 without mutation), and a moderate HCM (stage B2) was diagnosed in 12 cats (*n* = 6 heterozygotes; *n* = 2 homozygotes, age at onset 2.5 years and 5.9 years; *n* = 4 without mutation). Severe HCM (stage C) was found in eight cats, of which three were diagnosed with CHF.

During the study period, CHF developed in four additional cats with severe HCM (*n* = 3 homozygotes, age at onset 11.7 years, 9.8 years, and 6.3 years; *n* = 1 without mutation, 12.2 years). In four cats with SAM (*n* = 2 heterozygotes, age at onset 7.8 and 11.4 years; *n* = 1 homozygote, 5.9 years; *n* = 1 without mutation, 8.3 years), the disease developed from a moderate to severe stage.

## 4. Discussion

The prognosis of HCM usually depends on the stage of the disease. Many cats with mild to moderate HCM never progress to severe HCM; hence, they have an excellent prognosis [17]. However, with consistent follow-up, a significant number of cats can develop severe HCM. In our study, 30.4% (7/23 cats) progressed to a severe stage of HCM with the development of CHF. Furthermore, 13% of cats with pronounced LV wall thickening and moderate to severe left atrial enlargement (stage B2), who did not suffer from heart failure, developed CHF. Factors associated with an increased risk of worsening and developing CHF were moderate to severe LA enlargement, decreased LV systolic function, available gallop rhythm or arrhythmia, prolonged IVRT time, higher body weight, homozygous genotype, and age greater than 6 years.

As noted in this study, the disease developed more severely in males than in females and often earlier (on average 1.7 years in males versus 2.8 in females). For example, there were cats with moderate HCM for 3 years and cats that had disease progressing to a severe degree, whereas others had HCM that had not manifested for several years. Judging by the absence of HCM at the age of up to 1 year and its appearance at a later age, the disease had a clear age-related penetrance.

Cough was detected in only five cats in the cardiomyopathy group. There was a significant difference in the groups when accounting for heart murmurs (*p* < 0.001), in the CM group, 50 cats had a heart murmur in the medical history, and 33 cats with HCM and dynamic LVOT obstruction had grade 3/6 murmurs, but cats with restrictive cardiomyopathy had no murmurs. However, in the congenital heart disease group, heart murmurs were present in all 10 cats, ranging from grade 3/6 to 5/6. This suggests that coughing is unlikely in cats with HCM. It can also be stated that the percentage of heart murmurs in cats with HCM is consistent with previously published data [18].

Anterior systolic mitral motion, causing dynamic left ventricular outflow tract obstruction, is common in cats with HCM. SAM occurs as a result of hypertrophied and cranially displaced papillary muscles that pull part of the mitral valve leaflet into the normal or narrowed left ventricular outflow tract, where at least part of the leaflet is in contact with the base of the interventricular septum. When the mitral valve leaflets are distorted because of SAM, mitral regurgitation also occurs, which can cause increased left atrial pressure. This results in a characteristic bilobed color Doppler jet with simultaneous systolic turbulence in the aorta and left atrium. SAM is a common cause of heart murmurs in cats with HCM. The murmur is often dynamic, meaning it gets louder when the cat is excited/stressed and quieter when the cat is relaxed. This occurs because physical or emotional stress increases the severity of SAM, which in turn increases the flow rate through dynamic LV outflow tract obstruction [19].

According to this study, anterior systolic mitral motion was detected in nine cats with severe to moderate HCM, which was also demonstrated by loud heart murmur. SAM is a common cause of heart murmur in cats with HCM. The noise is often dynamic, meaning it gets louder when the cat is excited/stressed and quieter when the cat is relaxed. This occurs because physical or emotional stress increases the severity of SAM, which in turn increases the flow rate through dynamic LV outflow tract obstruction [20].

The gallop rhythm in small animals (S3 and S4) is usually associated with impaired diastolic function. Some third heart sounds in cats are nonspecific, complicating their classification based on auscultation alone. A third heart sound that is sometimes present and sometimes absent on a single auscultation in sinus rhythm (e.g., a third heart sound is present, then resolves, then repeats, all within 1 min or less). It is unlikely to be an S3 or S4 gallop rhythm, but it is more likely to be a systolic click because the first sounds indicate high diastolic ventricular filling pressure, which is not expected to cycle from audible to inaudible and back again within a short time (only a few seconds) [21].

In this study, gallop rhythms were present in 31% of cats with HCM (*n* = 7). Heart murmurs, mostly of grade 3/6, were present in 60% of cats with HCM (*n* = 14), and 9% of cats (*n* = 2) had a grade 4/6 murmur. In 13% (*n* = 3) of cats, there were no heart sounds on auscultation. A parasternal systolic heart murmur is reported in 80% of cats with subclinical HCM, compared to 30–45% of healthy cats without HCM. Third heart sounds such as a gallop rhythm are noted in 2.6–19% of cats with subclinical HCM and are rarely present in healthy cats. Arrhythmias may also be associated with cardiomyopathies, although many affected cats do not have auscultatory abnormalities. A loud systolic murmur (grade 3–4/6) is more common in cats with HCM than in healthy cats, but an increase in the intensity of the heart murmur over time does not necessarily indicate the presence or worsening of the disease [22].

The following arrhythmias were revealed in the group of cats with cardiomyopathies: sinus tachycardia was found in 8/77 cats, atrial fibrillation in 7/77 cats, and ventricular arrhythmia in 14/77 cats. In the “Cardiomyopathies” group, a greater predisposition of males to HCM was confirmed (*n* = 41). It can be concluded that with increasing severity of the disease, the frequency of arrhythmias grew. Resting ECG can detect numerous types of arrhythmias, including premature atrial and ventricular contractions (PACs and PVCs), atrial and ventricular tachycardia, and atrial fibrillation. Although ECG is sensitive for detecting atrial fibrillation, the diagnosis of atrial fibrillation in cats is not easy (the ECG may not be specific for feline atrial fibrillation). Sinus rhythm with small P waves (which may be obscured by artifacts), rapid PVCs, and atrial tachycardia are sometimes masked as AF in cats [19].

In this study, it was noted that all cats with severe HCM had increased left atrial diameter and prolonged isovolumic relaxation time. However, as one publication [23] showed, not all cases of left-sided heart failure may be manifested with left atrial and ventricular enlargement.

According to the research results, it was established that, regardless of the breed and age category, the morphological blood parameters in cats were within the physiological norm, whereas the hematocrit in three cats (13%) was below normal. However, this was not associated with disease severity. It was established that the biochemical profile indicators in cats with HCM were within the physiological norm. At the same time, there was a slight increase in the level of liver enzymes (ALT and AST) in 12 of 23 cats (52%), and a moderate increase in creatinine and urea in 4 cats (17%), which was not associated with disease severity.

The research results revealed that diagnostic methods such as ECG and chest X-rays appear to be quite specific but less sensitive indicators of HCM. A normal P wave on an ECG and a normal cardiac silhouette on chest films do not reliably exclude hypertrophic cardiomyopathy in an individual cat. Both diagnostic methods cannot be used as clinically valid substitutes for echocardiography in the qualitative and quantitative assessment of left ventricular size in cats.

Accurate prediction of disease outcome based on genetic tests alone seems an unlikely task [24]. However, the importance of performing a genetic test is significant because five of the six homozygous cats in this study had an increase in disease severity over the study period.

## 5. Conclusions

Similar to human HCM, feline HCM [25] likely has multiple causes due to mutations in multiple genes and possible interactions with other genetic and environmental factors.

Thus, additional research is needed to better understand the molecular mechanism of the disease and the consequences of gene mutations, which are currently poorly understood. Feline HCM is a good model for human HCM because nearly 35% of human cases of HCM are associated with mutations in the *MYBPC3* gene [25], which have also been found in cats.

This study provides information on the clinical signs and differences in cardiac disease and the parameters of echocardiographic measurements in feline HCM, which may assist practitioners in diagnosis. According to our data, coughing is unlikely in cats with dyspnea as a result of CHF, and heart murmurs may be undetectable. The presence of gallop rhythm and arrhythmia is a significant sign of heart disease.

HCM can manifest itself in varying degrees of severity, and early diagnosis, based on a complex of clinical studies and DNA testing to identify missense mutations in the *MYBPC3* gene, can prevent complications associated with the disease, such as congestive heart failure due to severe diastolic dysfunction. Cats homozygous for the p. A31P mutation of the *MYBPC3* gene and cats older than 6 years had a more severe stage of HCM. This may indicate that cats with the identified p. A31P mutation (including heterozygous ones) and animals at a more mature age must undergo an annual cardiac examination compared with animals without this gene mutation and under the age of 5 years.

## Figures and Tables

**Table 1:** Results of examination of cats with diseases of the cardiovascular system.

Indicator	Cardiomyopathies, *n* = 77	Secondary Heart Diseases, *n* = 16	Congenital Heart Diseases, *n* = 10	Significance, *p*
Median age	8.5	14	1	<0.001
Age range	1–22	8–22	0.25–9	-
Males, (% of animals)	61	65	40	-
Median HR, bpm	190	200	190	-
Heart murmur, (% of animals)	65	94	100	<0.001
Gallop rhythm, (% of animals)	32	12.5	33	<0.001
Dyspnea, (% of animals)	68	56	50	<0.001
Cough, (% of animals)	6	19	0	<0.001
Arrhythmia, (% of animals)	35	37.5	40	<0.001

**Table 2:** Incidence of HCM among heart diseases in cats of different breeds.

Breed	Total (*n* = 103)	Cardiomyopathies (*n* = 77)	Incl. Cats with HCM (*n* = 61)	Congenital Heart Diseases (*n* = 10)	Secondary Heart Diseases (*n* = 16)
Domestic Shorthair	42	32	20	3	7
Domestic Longhair	18	9	8	1	8
Abyssinian	1	1	1	0	0
Bengal	2	0	0	2	0
British Shorthair	10	6	6	3	1
Maine Coon	18	18	18	0	0
Norwegian forest	3	3	3	0	0
Persian	3	3	3	0	0
Siamese	1	1	0	0	0
Siberian	2	2	0	0	0
Sphinx	2	2	2	0	0
Scottish lop-eared	1	0	0	1	0

**Table 3:** Age penetrance of feline HCM (*n* = 61).

Age	Percentage, %
1–3 years	21.3 (*n* = 13)
4–5 years	34.4 (*n* = 21)
>7 years	44.3 (*n* = 27)

**Table 4:** Predisposition of cats to HCM by sex.

Sex	Number of Animals	Percentage, %
Females	20	32.8
Males	41	67.2
Total	61	100

**Table 5:** Results of hematology laboratory tests.

Test Name, Units	Normal Range	Result
RBC, ×10^12^/L	5.3–10.0	10.1 ± 0.7
HGB, g/L	80–170	147.02 ± 0.14
HCT, %	26–48	31.04 ± 2.07
WBC, Total, ×10^9^/L	5.5–18.5	17.9 ± 1.12
PLT, ×10^9^/L	300–630	467.06 ± 17.02

RBC, red blood cell count; HGB, hemoglobin; HCT, hematocrit; WBC, white blood cell count; PLT, platelet count.

**Table 6:** Results of biochemistry laboratory tests.

Test Name, Units	Normal Range	Result
Urea, mmol/L	4.0–8.0	7.1 ± 0.73
Creatinine, umol/L	50.0–160.0	156.9 ± 4.91
Total bilirubin, umol/L	0.0–8.0	7.3 ± 0.76
AST, U/L	9.0–39.5	29.8 ± 1.71
ALT, U/L	17.0–79.0	79.3 ± 3.42
Glucose, mmol/L	3.2–6.4	4.1 ± 0.38
Total protein, g/L	54.0–79.5	62.1 ± 2.31
Albumin, g/L	24.5–38.5	27.2 ± 1.66
Cholesterol, mmol/L	1.8–4.7	3.4 ± 0.74

AST, aspartate aminotransferase; ALT, alanine aminotransferase.

**Table 7:** Results of examination of cats with HCM.

Indicator	Cats w/o HCM (Mean Values)	Cats with HCM (Mean Values)
IVSd (cm)	0.42 ± 0.04	0.64 ± 0.10
LVWd (cm)	0.40 ± 0.04	0.63 ± 0.11
LVd (cm)	1.68 ± 0.22	1.69 ± 0.13
LVs (cm)	0.81 ± 0.15	0.78 ± 0.06
FS (%)	52.45 ± 4.79	53.78 ± 3.32
Ao (cm)	0.95 ± 0.13	1.08 ± 0.07
LA (cm)	1.16 ± 0.18	1.50 ± 0.29
LA/Ao	1.23 ± 0.13	1.39 ± 0.24
Fl Ao—Vmax (m/s)	1.06 ± 0.23	2.58 ± 1.85
Fl PA—Vmax (m/s)	1.04 ± 0.16	0.96 ± 0.14
E wave (m/s)	0.93 ± 0.20	0.93 ± 0.38
A wave (m/s)	0.59 ± 0.15	0.75 ± 0.10
E/A	1.38 ± 0.27	1.03 ± 0.30
IVRT	54.09 ± 7,4	71.1 ± 16.2

IVSd, interventricular septum in diastole; LVWd, left ventricular wall in diastole; LVs, left ventricle in systole; LVd, left ventricle in diastole; FS, fractional shortening; Ao, aorta; LA/Ao, left atrial-to-aortic ratio; LA, left atrium; Fl Ao—Vmax, maximum velocity of aortic blood flow; Fl PA—Vmax, maximum blood flow velocity in the pulmonary artery; E wave, early LV filling speed; A wave, LV filling velocity during atrial systole; E/A, peak velocity of the early diastolic wave-to-peak velocity of the late diastolic wave ratio; IVRT, isovolumic relaxation time.

**Table 8:** Age distribution of genotypes by severity.

Age		Heterozygous, *n* = 8 (% of Animals)	Homozygous, *n* = 6 (% of Animals)	Without Mutation, *n* = 9 (% of Animals)
1–3 years	mild	4.3	-	4.3
	moderate	8.7	4.3	4.3
	severe	-	-	-
4–6 years	mild	-	-	4.3
	moderate	8.7	4.3	-
	severe	-	4.3	8.7
>7 years	mild	-	-	-
	moderate	8.7	-	13
	severe	4.3	13	4.3

## Data Availability

All data generated for this study are presented within the manuscript.
